# Supplementary material for: Controlling the dimension of the quantum resonance in CdTe quantum dot superlattices fabricated via layer-by-layer assembly
Source: Nat Commun. 2020 Oct 29;11:5471. doi: 10.1038/s41467-020-19337-0 (PMC7596095; doi:10.1038/s41467-020-19337-0)
Supplement: Supplementary file 1 — Supplementary Information [file 41467_2020_19337_MOESM1_ESM.pdf]

Supplementary Information

Controlling the dimension of the quantum resonance in CdTe quantum dot superlattices fabricated via layer-by-layer assembly

TaeGi Lee[1], Kazushi Enomoto[2], Kazuma Ohshiro[1], Daishi Inoue[2], Tomoka Kikitsu[2], Kim Hyeon-Deuk[3], Yong-Jin Pu[2]*, and DaeGwi Kim[1]*

Correspondence to: tegi@a-phys.eng.osaka-cu.ac.jp; yongjin.pu@riken.jp

Supplementary Note 1

Supplementary Figure 1a shows a TEM image of the typical CdTe QDs used in this study. Supplementary Figure 1b shows a histogram obtained by measuring the diameters of 50 QDs from the image. The solid curve represents a Gaussian fitting function with an average size of 3.4 nm and a standard deviation of 0.3 nm.

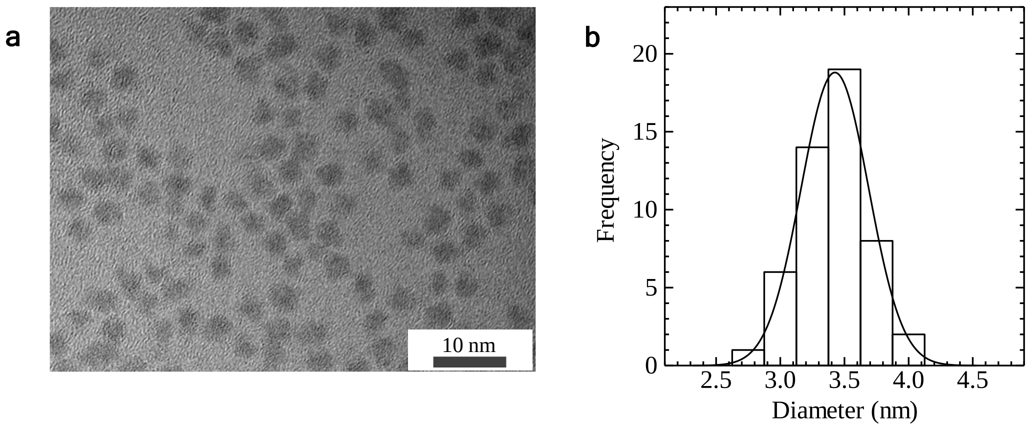

Supplementary Figure 1 | TEM image of CdTe QDs synthesised via the hydrothermal method. **a,** TEM image of CdTe QDs. **b,** Size histogram of the CdTe QDs. The solid curve represents a Gaussian fitting function with an average size of 3.4 nm and a standard deviation of 0.3 nm.

Supplementary Note 2

The extinction spectra of CdTe QD monolayer and 3 layers prepared under the condition of OD_{sol} = 0.20 are shown in Supplementary Fig. 2a and 2b, respectively. Focusing on the energy region lower than 2.0 eV, it can be seen that the intensity of the extinction spectra is not flat. In other words, there is a baseline. Supplementary Figure 2c shows the extinction spectra of PDDA/PAA multilayers without CdTe QDs and the extinction spectra of ZnS QD multilayers. Here, ZnS QDs are completely transparent in the photon energy region of 1.75 to 2.75 eV, which is the focus of attention on the absorption properties of CdTe multilayers in this study. Both normalized extinction spectra are shown in Supplementary Fig. 2d. It is clear that the two normalized extinction spectra are in good agreement. Furthermore, the extinction spectrum shown in Supplementary Fig. 2d also coincides with the baseline in the extinction spectra of the CdTe monolayer and 3 layers in Supplementary Figs. 2a and 2b. These facts mean that this baseline does not reflect the 'absorption' of CdTe QDs but reflects the influence of light scattering due to the polymer layer and/or the stacking QDs. Therefore, we obtained the absorption spectra specific to CdTe QD multilayers from the extinction spectra by correcting the baseline caused by scattering. Then, the absorption peak energy was evaluated from the obtained absorption spectra. Supplementary Figures 2e and 2f show the normalized extinction and absorption spectra of CdTe QD monolayer and 3 layers prepared under the condition of OD_{sol} = 0.20. From both the figures, the low-energy shifts of the absorption peak due to the quantum resonance are clearly observed.

To discuss the experimental results for sub-monolayers, the extinction spectra of CdTe QD monolayer prepared under the condition of OD_{sol} = 0.02 and 0.20 are shown in Supplementary Figs. 3a and 3b, respectively. The peak structure is clearly observed even in the extinction spectrum of the sub-monolayer. Furthermore, it can also be seen that the influence of the baseline on the sub-monolayer is small. The normalized extinction spectrum before the baseline correction and the absorption spectrum after the correction are shown in Supplementary Figs. 3c and 3d. It is evident that the peak energy is almost unchanged before and after the baseline correction.

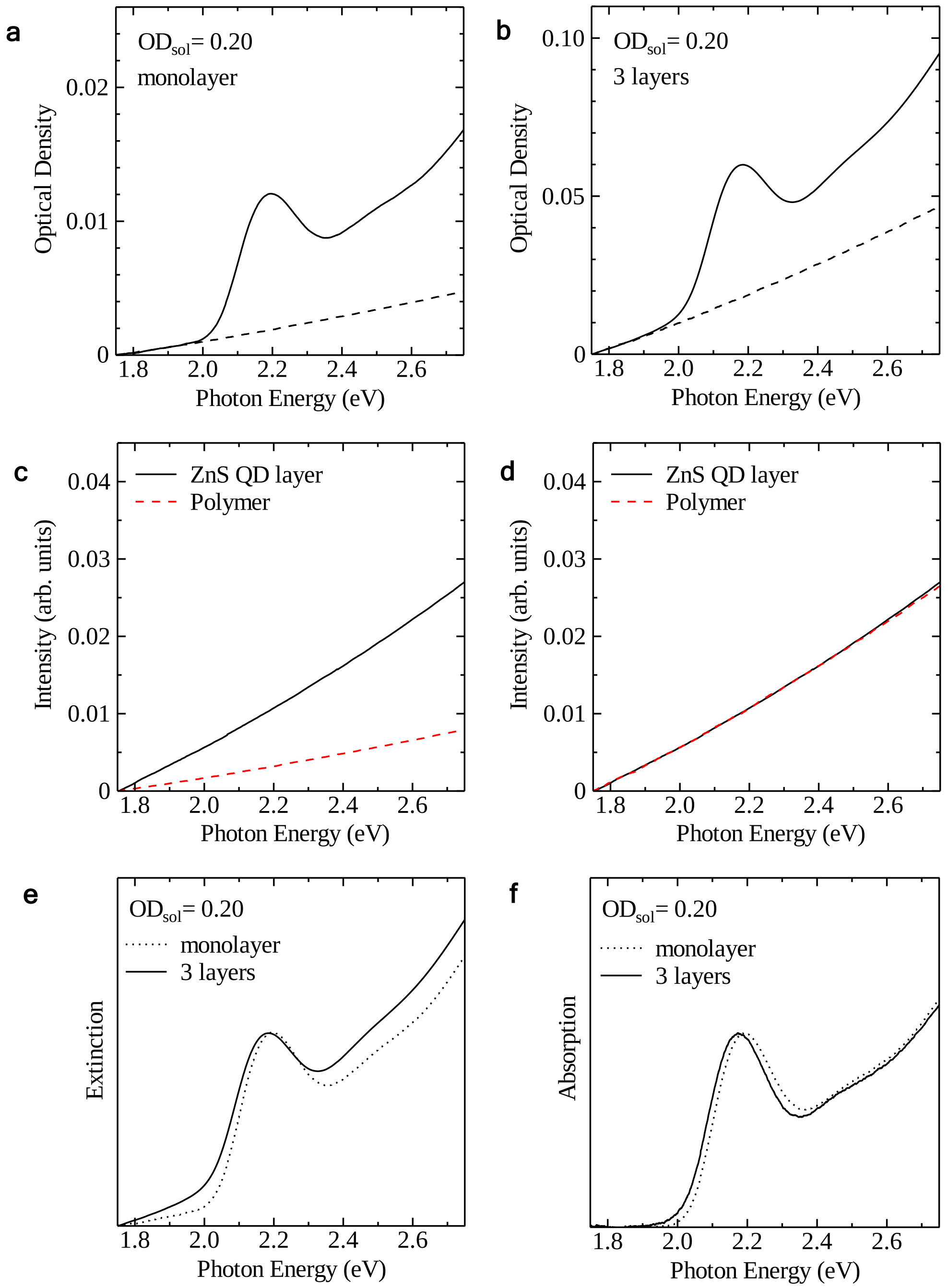

Supplementary Figure 2 | Analysis of absorption spectra of CdTe QD monolayer and 3 layers with high $\sigma_{\text{in-plane}}$. a,b, The extinction spectra of CdTe QD (a) monolayer and (b) 3 layers prepared under the condition of OD_{sol} = 0.20. The dashed curves show the baseline in the extinction spectra. **c,d,** (c) the extinction spectra of PDDA/PAA multilayers without CdTe QDs and the extinction spectra of ZnS QD multilayers and (d) the normalized spectra. **e,f,** the normalized (e) extinction spectra and (f) absorption spectra, which are corrected, of CdTe QD monolayer and 3 layers prepared under the condition of OD_{sol} = 0.20.

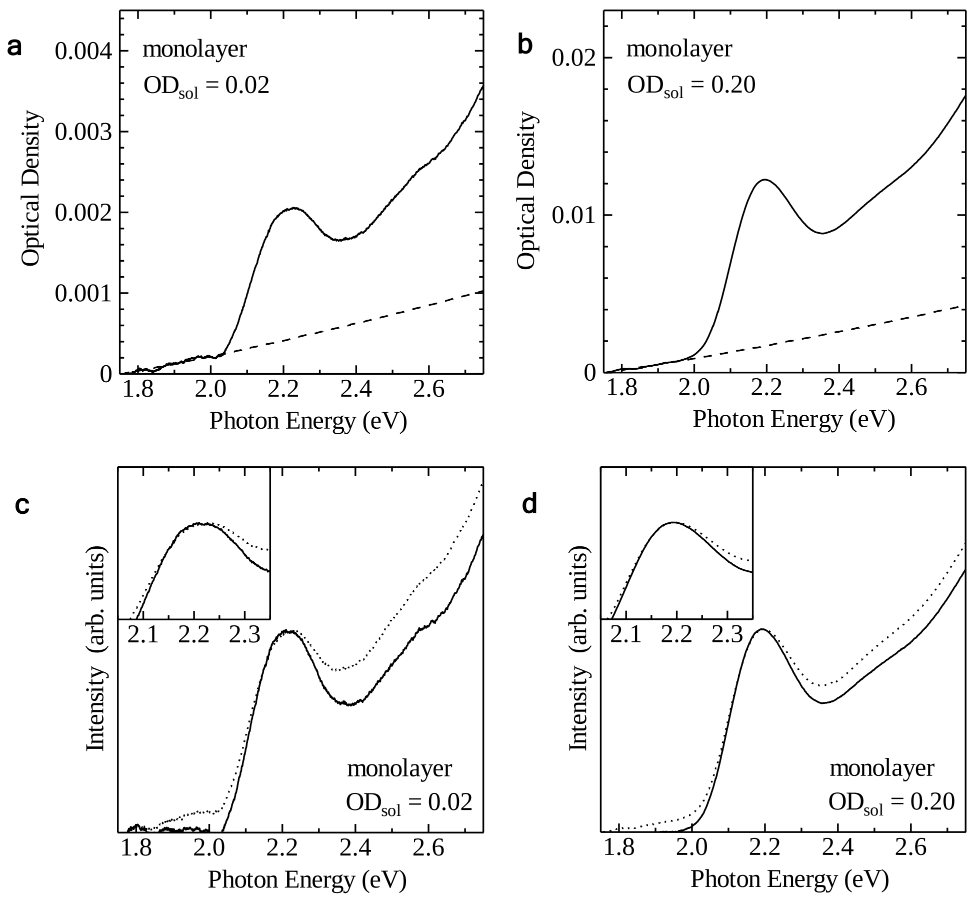

Supplementary Figure 3 | Analysis of absorption spectra of CdTe QD monolayers with low and high $\sigma_{in\text{-}plane}$. a,b, The extinction spectra of CdTe QD monolayers prepared under the condition of (a) OD_{sol} = 0.02 and (b) OD_{sol} = 0.20. The dashed curves show the baselines in the extinction spectra. **c,d,** The dashed and solid curves show the normalized extinction spectra and the absorption spectra, which are corrected, respectively, of the QD monolayers prepared under the condition of (c) OD_{sol} = 0.02 and (d) OD_{sol} = 0.20. The insets are the enlarged figures in the energy region near the peak.

Supplementary Note 4

It was revealed that $\Delta E_{\text{in-plane}}$, which is the energy shift due to the in-plane quantum resonance, is 19 meV. In addition, the energy shift $\Delta E_{\text{in-plane}}$ owing to the quantum resonance in the stacking direction is 22 meV and 20 meV in the QD multilayers prepared with low and high $\sigma_{\text{in-plane}}$, respectively. The difference between the absorption energies of isolated QD sample and 3D QDSL is 39 meV, which corresponds to the sum of the resonant coupling energies due to the quantum resonance in both the stacking and in-plane direction.

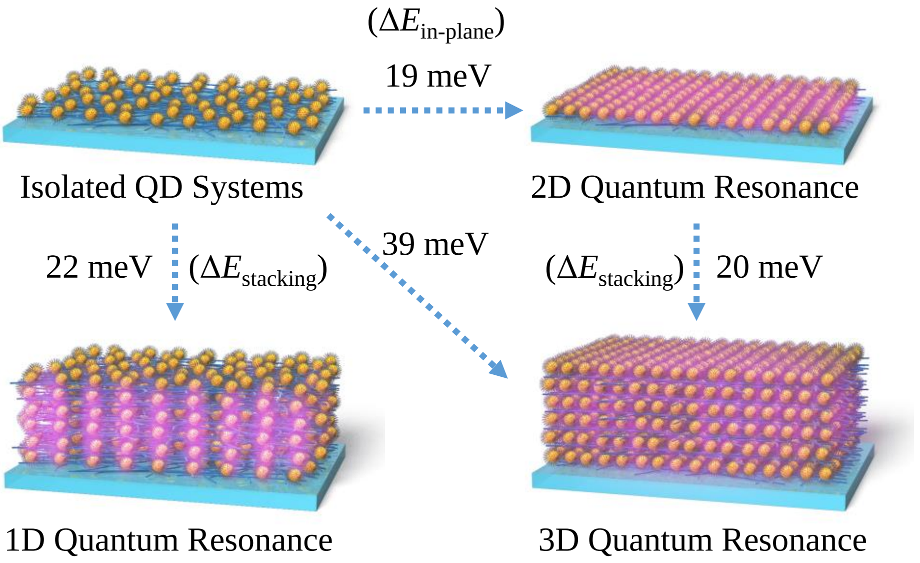

Supplementary Figure 4 | Schematics of QD layer structures in which the 1D, 2D, and 3D quantum resonances occur, and the energy shift of the absorption peak due to the quantum resonance. The difference of the absorption peak energies between the QD monolayer with high $\sigma_{\text{in-plane}}$ and the QD monolayer with low $\sigma_{\text{in-plane}}$ ($\Delta E_{\text{in-plane}}$) is 19 meV. The difference in the absorption peak energies between the QD monolayer with n = 1 and QD multilayer with n = 5 ($\Delta E_{\text{stacking}}$) is 22 meV and 20 meV in the samples with low and high $\sigma_{\text{in-plane}}$, respectively.

Supplementary Note 5

Supplementary Figure 5a shows TEM images of the OA-capped CdTe QDSL. The average diameter of the OA-capped CdTe QDs is 4.2 nm, with a standard deviation of 0.4 nm. Supplementary Figure 5b shows the excitation energy dependence of the PL spectra of OA-capped CdTe QD solution. The down arrows indicate the excitation energies in the PL measurements. The PL peak shifts towards lower energies as the excitation energy decreases. Supplementary Figures 5c and 5d show the results of the excitation energy dependence of the PL spectra and detection energy dependence of the PLE spectra in OA-capped CdTe QDSL, respectively. The PL peak and PLE peak are shifted, that is, the size selectivity of the optical properties is observed in OA-capped CdTe QDSL.

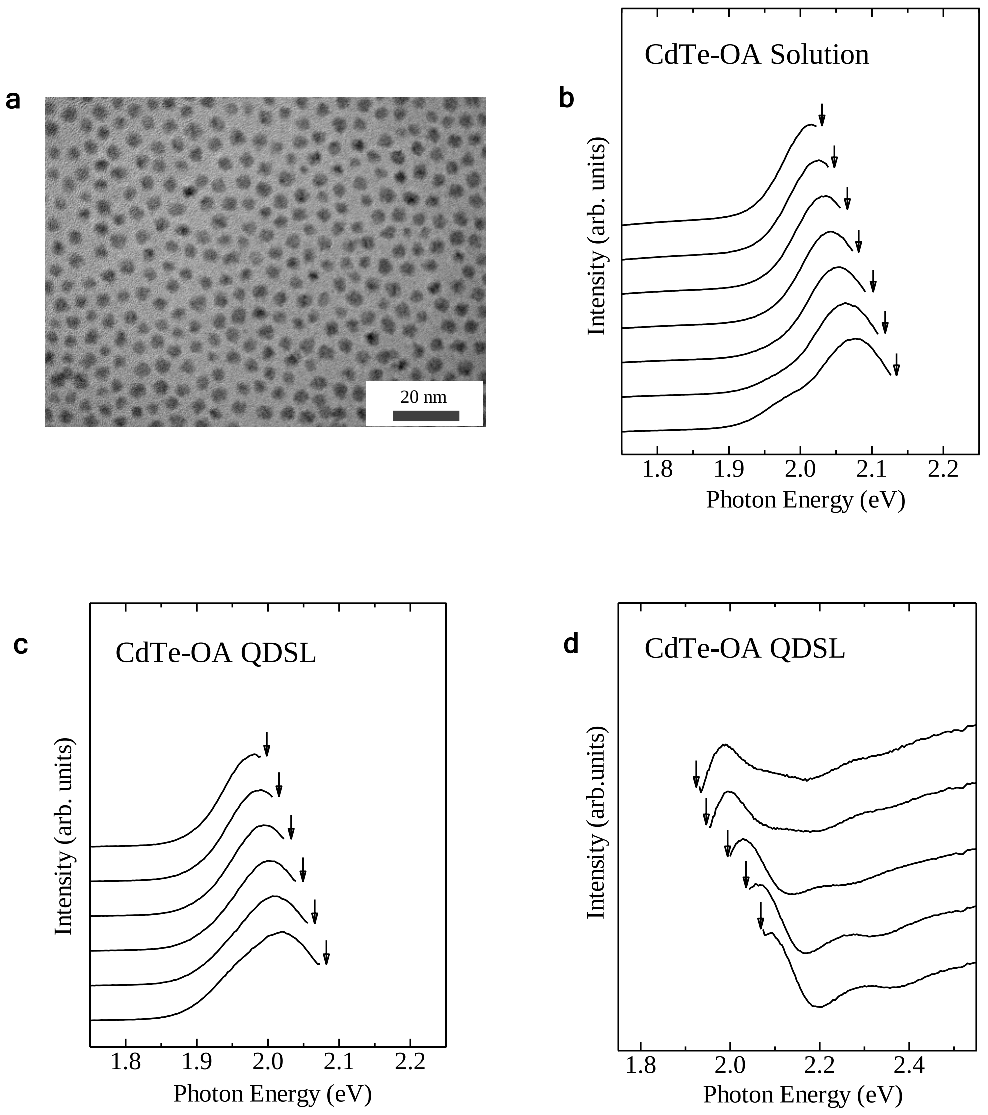

Supplementary Figure 5 | TEM image, excitation energy dependence of the PL spectra, and detection energy dependence of the PLE spectra of OA-capped CdTe QDSL. a, TEM image of OA-capped CdTe QDs. **b,c,** Excitation energy dependence of the PL spectra of OA-capped CdTe QD solution (b) and QDSL (c). The solid lines show PL spectra with different excitation energies. The down arrows indicate the excitation energy in the PL measurements. **d,** Detection energy dependence of the PLE spectra of OA-capped CdTe QDSL. The solid lines show PLE spectra with different detection energies. The down arrows indicate the detection energies in the PLE measurements.

Supplementary Note 6

Supplementary Figure 6a shows the PL-decay profiles of CdTe QDSL in which 1D quantum resonance occurs at 80 K, 180 K, and 250 K. The solid curves are the result of fittings performed using three exponential functions. The temperature dependence of the average decay time is shown in Supplementary Fig. 6b. In addition, we measured the temperature dependence of the PL decay time in the QDSLs in which 1D, 2D, and 3D (Supplementary Figs. 6b, 6c, and 6d, respectively) quantum resonances occur.

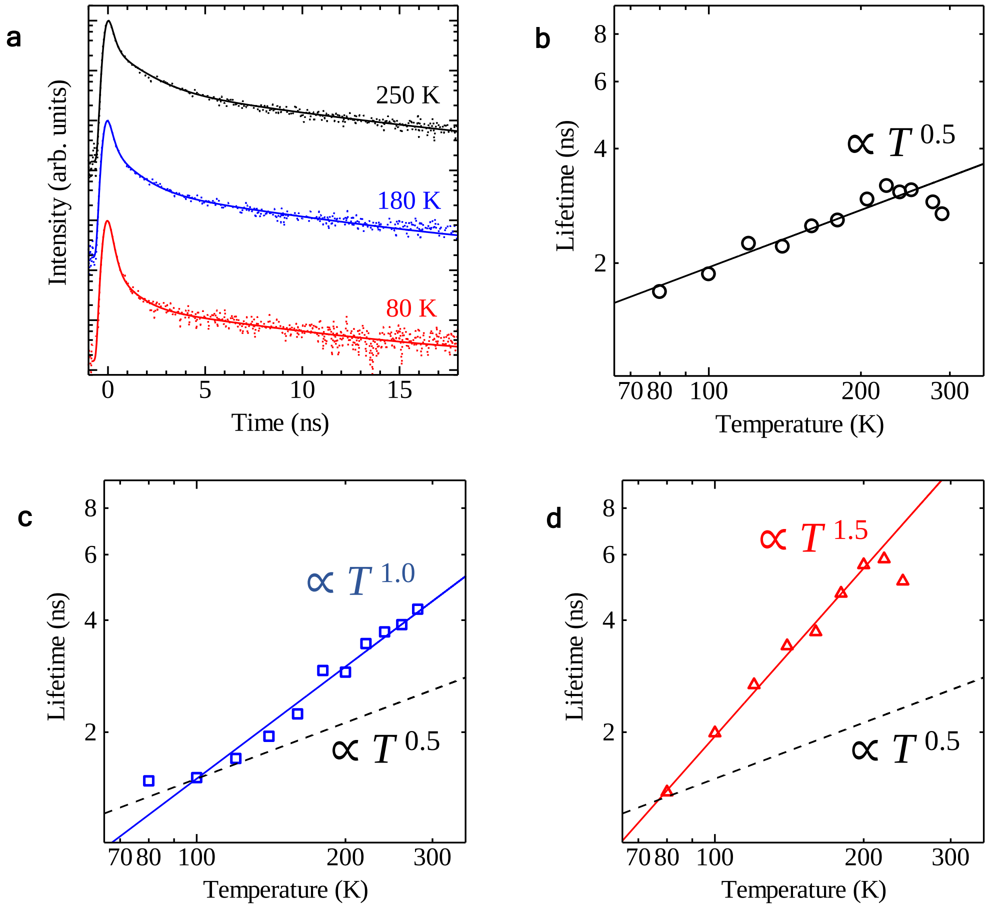

Supplementary Figure 6 | Temperature dependence of radiative lifetime in the QDSL in which 1D, 2D, and 3D quantum resonance occurs. a, The dotted lines are PL-decay profiles of the QD layer in which 1D quantum resonance occurs, which were measured at 80 K (red), 180 K (blue), 250 K (black), respectively. The solid curves are the results of fitting performed using three exponential functions. **b,** The open circles represent the average decay times at each of the temperatures of the QD layer in which 1D quantum resonance occurs; the solid line represents a $T^{0.5}$ dependence. **c,** The open rectangles represent the average decay times at each of the temperatures of the QD layer in which 2D quantum resonance occurs; the solid line represents a $T^{1.0}$ dependence, and dashed line represents a $T^{0.5}$ dependence. **d,** The triangles represent the average decay times at each of the temperatures of the QD layer in which 3D quantum resonance occurs; the solid line represents a $T^{1.5}$ dependence, and dashed line represents a $T^{0.5}$ dependence.
